# Morbidity profile and pharmaceutical management of adult outpatients between primary and tertiary care levels in Sri Lanka: a dual-centre, comparative study

**DOI:** 10.1186/s12875-024-02448-8

**Published:** 2024-06-06

**Authors:** Inosha Alwis, Buwanaka Rajapaksha, Chanuka Jayasanka, Samath D. Dharmaratne

**Affiliations:** 1 Department of Community Medicine, Faculty of Medicine, University of Peradeniya, Peradeniya, Sri Lanka; 2 Faculty of Medicine, University of Peradeniya, Peradeniya, Sri Lanka

**Keywords:** Reasons for encounter, Disease burden, Treatment patterns, Rational prescribing, Health system, South Asia

## Abstract

**Background:**

Outpatient care is central to both primary and tertiary levels in a health system. However, evidence is limited on outpatient differences between these levels, especially in South Asia. This study aimed to describe and compare the morbidity profile (presenting morbidities, comorbidities, multimorbidity) and pharmaceutical management (patterns, indicators) of adult outpatients between a primary and tertiary care outpatient department (OPD) in Sri Lanka.

**Methods:**

A comparative study was conducted by recruiting 737 adult outpatients visiting a primary care and a tertiary care facility in the Kandy district. A self-administered questionnaire and a data sheet were used to collect outpatient and prescription data. Following standard categorisations, Chi-square tests and Mann‒Whitney U tests were employed for comparisons.

**Results:**

Outpatient cohorts were predominated by females and middle-aged individuals. The median duration of presenting symptoms was higher in tertiary care OPD (10 days, interquartile range: 57) than in primary care (3 days, interquartile range: 12). The most common systemic complaint in primary care OPD was respiratory symptoms (32.4%), whereas it was dermatological symptoms (30.2%) in tertiary care. The self-reported prevalence of noncommunicable diseases (NCDs) was 37.9% (95% CI: 33.2–42.8) in tertiary care OPD and 33.2% (95% CI: 28.5–38.3) in primary care; individual disease differences were significant only for diabetes (19.7% vs. 12.8%). The multimorbidity in tertiary care OPD was 19.0% (95% CI: 15.3–23.1), while it was 15.9% (95% CI: 12.4–20.0) in primary care. Medicines per encounter at primary care OPD (3.86, 95% CI: 3.73–3.99) was higher than that at tertiary care (3.47, 95% CI: 3.31–3.63). Medicines per encounter were highest for constitutional and respiratory symptoms in both settings. Overall prescribing of corticosteroids (62.7%), vitamin supplements (45.8%), anti-allergic (55.3%) and anti-asthmatic (31.3%) drugs was higher in the primary care OPD, and the two former drugs did not match the morbidity profile. The proportion of antibiotics prescribed did not differ significantly between OPDs. Subgroup analyses of drug categories by morbidity largely followed these overall differences.

**Conclusions:**

The morbidities between primary and tertiary care OPDs differed in duration and type but not in terms of multimorbidity or most comorbidities. Pharmaceutical management also varied in terms of medicines per encounter and prescribed categories. This evidence supports planning in healthcare and provides directions for future research in primary care.

**Supplementary Information:**

The online version contains supplementary material available at 10.1186/s12875-024-02448-8.

## Background

According to the Declaration of Alma-Ata, primary care refers to comprehensive and essential health services delivered at the point of first contact [1]. Tertiary care includes facilities that provide highly specialised services involving advanced diagnostics and therapeutics. Outpatient care, health services delivered on an outpatient basis, are available at both these levels of the health system. There is global evidence on how primary care outpatient departments (OPDs) differ from tertiary care OPDs in multiple ways, including the morbidity profile of the attendees, their management and outcomes [2–4]. Analysing these morbidity and treatment patterns in outpatient care can inform evidence-based care processes and health planning [5, 6]. However, such comparisons between different levels of health systems in South Asia have been limited by temporal and geographic variations in the conducted studies [7–10]. Our study aimed to overcome this limitation by comparing a primary and a tertiary care outpatient setting in the region within a common time point and geography.

Sri Lanka is an island nation in South Asia with a population of 21 million. It is globally recognised for its low-cost, high-impact health system [11]. Annually, about fifty million public-sector outpatient consultations take place in Sri Lanka. Despite this number of visits exceeding twice the country’s population, there is currently no national surveillance mechanism to capture outpatient morbidity or management [12]. The Sri Lankan literature on outpatient morbidity has thus far examined tertiary care settings, with no published studies on public sector primary care [9, 13]. This study aims to address these crucial gaps in knowledge.

Out of outpatient visits in Sri Lanka, more than half are made to primary care OPDs [14]. Over ten million visits are also made to tertiary care OPDs, with patients often selecting to bypass primary care gatekeepers to seek help in tertiary care [14, 15]. Therefore, observing the differences between primary and tertiary care OPDs in Sri Lanka can provide important implications of the bypassing phenomenon that is not uncommon in low- and middle-income (LMIC) countries [16].

Despite the epidemiological transition seen in many South Asian countries, there is limited evidence that compares and contrasts the noncommunicable disease (NCD) status of outpatients between different levels of care. Evidence is also lacking about the multimorbidity of outpatients between different levels of care, which is becoming a major challenge with the ageing populations across the world.

Polypharmacy is a growing concern to many health systems worldwide. This is also true for Sri Lanka, as the average number of medicines per patient encounter in tertiary care settings was observed to be higher than the World Health Organisation’s (WHO) recommended value [17]. However, evidence is lacking on the status of polypharmacy in primary care OPDs in Sri Lanka. Furthermore, generating evidence on commonly prescribed medicines at different levels in the health system is essential for health management as well as for outcome-based medical education [17].

Rational prescribing is a crucial goal for LMIC health systems. Most studies performed to assess rational prescribing patterns in outpatient care have focused on antibiotic usage. However, the misuse of other medicines in outpatient care, such as nonsteroidal anti-inflammatory drugs (NSAIDs), multivitamin supplements and oral corticosteroids, has been reported in South Asia and worldwide [18, 19]. This study intended to explore these gaps in relation to different levels of care in the health system.

So, the main aim of this study was to describe and compare the morbidity profile and pharmaceutical management of adult outpatients between a primary and a tertiary care OPD in Sri Lanka.

## Methods

### Study design and setting

This was a comparative study conducted at two OPD settings in the Kandy district in Sri Lanka. Ethical approval was obtained from the Ethical Review Committee of the Postgraduate Institute of Medicine, Colombo (ERC No: ERC/PGIM/2022/103). Kandy district has the second-highest number of OPD visits in the country and houses a population of approximately 1.3 million people [14]. Two tertiary care settings in the Kandy district provide adult healthcare. One of them (TC) was selected as the tertiary care outpatient setting since the morbidity profile of the other tertiary care setting had already been assessed [9]. The average OPD admission rate at TC is approximately 1000 outpatients per day, and approximately 10 medical officers work during one OPD session. A primary care facility in a suburb in the Kandy district (PC) was selected as the primary care outpatient setting due to the availability of services and facilities that were standard for the primary care level in Sri Lanka. PC serves an outpatient population that is largely comparable to TC. The average OPD admission rate ranges from 200 to 400 outpatients per day, and one OPD session would be attended by a minimum of two medical officers.

### Study population and sample selection

Adult outpatients above 18 years of age who were visiting the outpatient departments at TC and PC during October 2022 were included in the study. Outpatients who were critically ill or who were registered at the OPD for the purposes of inward admission or transfer to other facilities were excluded. A minimum sample size of 730 was calculated using G*Power software (version 3.1.9.4) for a chi-square test that can estimate even a small effect size (approximating 0.1) to one degree of freedom [20]. The primary outcome was the proportion of outpatients with a given type of systemic complaint in the primary or tertiary care setting. The study was powered at 80%, allowing an alpha error of 5%, and the required sample size was inflated by 10% to account for nonresponse, rendering a final sample size of 803. Simple random sampling was used to select three random days per week to visit the OPD, and consecutive sampling was used to select outpatients at each setting until the final sample size was achieved. Recruitment at the primary care OPD was followed by tertiary care.

### Study instruments and data collection

This study used two main study instruments: a self-administered, structured questionnaire and a structured datasheet. The questionnaire contained items on sociodemographic variables (age, sex, ethnicity, resident area) and morbidity profile (presenting symptoms, duration, comorbidities). Multiple choices under the ‘presenting symptom’ item were developed by reviewing the local literature and were organized according to the broader categories in the International Classification of Primary Care Edition 2 (ICPC- 2) [9, 21, 22]. Some symptoms were amalgamated into a single presenting complaint (e.g., common cold) based on the literature. The questionnaire was translated into Sinhala and Tamil languages, pretested among a cohort of outpatients at another tertiary care facility in the Kandy district and assessed for face validity. The structured datasheet consisted of six columns under the headings of OPD registration number, name of the drug, dosage, frequency, duration and other remarks. Two pre-intern medical graduates were recruited and trained for data collection. Data collection was carried out in two stages. In the first stage, following introductions and informed consent, the principal investigator and data collectors administered the questionnaire to outpatients while they were in the waiting area of the OPD. In the second stage, prescriptions issued for outpatients were obtained from the OPD pharmacy, and details of the prescribed medicines were copied onto the structured datasheet.

### Data analysis

Data entry and data cleaning were performed using Microsoft Office Excel for Microsoft 365 MSO (Version 2203). Statistical Package for Social Sciences (SPSS Version 25.0) software was used for data coding and analysis. In the analysis of the presenting symptom, the percentages were initially calculated for each specific presenting symptom and then recalculated for the relevant systemic complaint according to ICPC-2 Chapters [22]. Multimorbidity was operationalised as the existence of two or more chronic conditions in the same individual [23]. In the analysis of the types of medicines, the medicines written by brand names were first converted to generic names, and the percentage of each type of generic medicine was calculated. Next, they were organized according to the categories of medicines in the National List of Essential Medicines 2014 [24]. The overall differences in medicines per encounter and percentages of prescribed medicine categories between the two settings were assessed first. This was followed by subgroup analyses of the number and categories of medicine based on the type of systemic complaint for both settings.

Independent sample t test was used to compare the means of normally distributed continuous variables between the two OPDs. Chi-square test was used to compare dichotomous categorical variables between the two OPDs. Multiple groups were recoded into dichotomous groups when required to make meaningful comparisons. Yates’s correction was applied during the subgroup analyses to compare proportions with smaller sample sizes. Kolmogorov‒Smirnov test was used to test the normality of distributions. To compare the distributions that were not normally distributed, Mann‒Whitney U test was applied. Probability value (*p*) < 0.05 was used as the critical value to decide if a result was statistically significant. Confidence intervals were calculated for selected key summary estimates.

## Results

### Participants

The total sample comprised 737 participants. The response rate was 90.8%. The majority were females (61.7%), and the mean age was 49.4 years (SD: 15.9 years). Most participants (39.2%) were middle-aged (40–59 years), 32.4% were older persons (60 years and above), and 8.4% were young persons (18–24 years). The majority of participants were Sinhalese (85.6%), followed by Muslims (8.6%), Tamils (5.6%) and Burghers (0.3%).

Three hundred and fifty-two outpatients were recruited at PC, while 385 participants were recruited at TC. The sociodemographic profile of outpatients at PC and TC is displayed in Table 1. As shown below, there were no statistically significant differences in the sex or age of outpatients between the two settings. There was a statistically significant difference in the ethnic composition of outpatients between primary and tertiary care.

**Table 1:** Sociodemographic characteristics of outpatients at primary and tertiary care OPDs

Sociodemographic characteristic		Primary Care OPD (PC)	Tertiary Care OPD (TC)	Statistical significance
		Number (%/SD)	Number (%/SD)	
Sex	Male Female	137 (38.9) 215 (61.1)	145 (37.7) 240 (62.3)	χ2 = 0.12, *p* = 0.72
Age	Mean age (years)	49.9 (16.2)	48.8 (15.5)	t = 0.986, *p* = 0.35
Age	Young (18–24 years) Adult (25–39 years) Middle-aged(40–59 years) Older persons (60 years &above)	24 (6.8) 74 (21.0) 133 (37.8) 121 (34.4)	38 (9.9) 73 (19.0) 156 (40.5) 118 (30.6)	χ2 = 3.56, *p* = 0.31
Ethnicity	Sinhalese Muslims Tamils Burgher	292 (83.0) 46 (13.1) 12 (3.4) 2 (0.6)	339 (88.1) 17 (4.4) 29 (7.5) 0 (0)	χ2 = 24.47, *p* < 0.001

SD – Standard deviation, χ2 – Chi-square statistic, t – t statistic

### Morbidity profile

#### Presenting symptoms

Outpatients presented to PC with 34 different types of presenting symptoms. The most common presenting symptom was common cold (23.6%), followed by wounds (8%), joint pains (7.4%), back pain (6.5%), headache (6.5%) and fever (6.5%).

At TC, outpatients presented with 38 different types of presenting symptoms. The most common presenting symptom was complaints related to skin (13.5%), followed by common cold (12.7%), wounds (8.8%), joint pains (8.1%) and back pain (5.7%).

#### Duration of presenting symptoms

Distributions of symptom duration at PC and TC were both positively-skewed. The median duration of the presenting symptom of outpatients at primary care OPD was three days (IQR:12), while it was 10 days (IQR:57) at tertiary care. Kolmogorov-Smirnov test showed a non-normal distribution (*p* < 0.001). The difference in the presenting symptom duration between the primary and tertiary care OPD was significant (U = 49748.5, *p* < 0.001).

Outpatients in primary care OPD had more acute symptoms (less than 3 days) than those in tertiary care (51.1% vs. 31.2%), and this difference was statistically significant (χ2 = 30.37, *p* = 0.00001). The outpatients at tertiary care OPD had a statistically higher prevalence of chronic symptoms (more than 1 month) compared to primary care (29.4% vs. 12.5%) (χ2 = 39.68, *p* = 0.00001).

### Systemic complaints

**Fig. 1:**
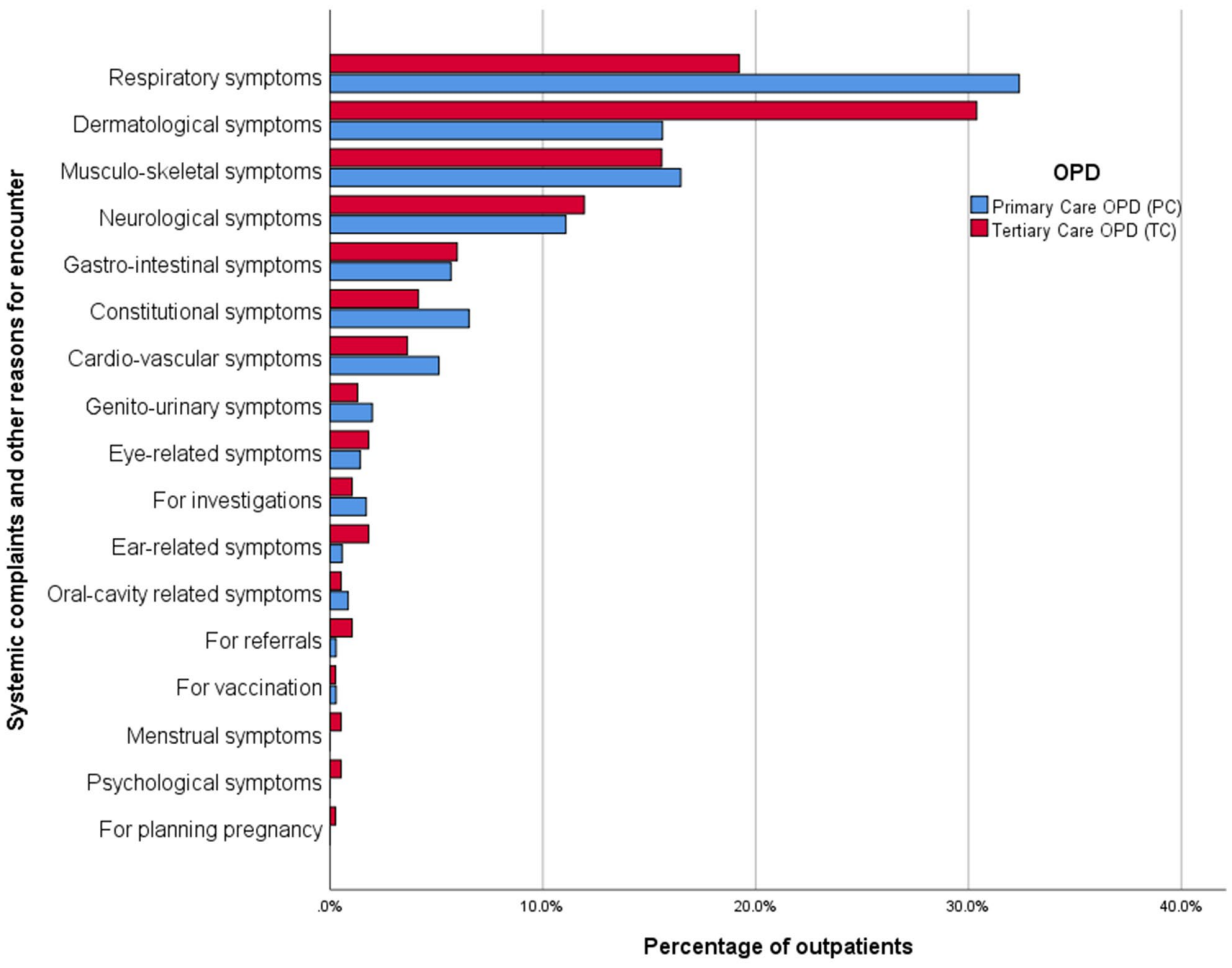
Systemic complaints of outpatients at primary and tertiary care OPDs

As illustrated in Fig. 1, the most common systemic complaint at the primary care OPD was respiratory symptoms (32.4%), followed by musculo-skeletal symptoms (16.5%) and dermatological symptoms (15.6%). The most common systemic complaint in the tertiary care setting was dermatological symptoms (30.4%), which included complaints related to skin, wounds, animal bites and other accidental trauma to skin according to ICPC-2 classification. This was followed by respiratory symptoms (19.2%) and musculo-skeletal symptoms (15.6%).

Among other reasons for encounter, visiting to perform investigations was the most common reason for both OPDs (1.7% at PC and 1% at TC). The proportion of psychological symptoms at both OPDs was less than 1%.

The systemic complaints were disaggregated by sex, age and ethnicity of the outpatients. As evident in Table 2, the most common systemic complaint in a given OPD setting remained the same across these sociodemographic variables. Nevertheless, the only exception was the prominence of musculo-skeletal symptoms (28.1%) among older persons visiting the primary care OPD, which was higher than respiratory symptoms (18.2%).

**Table 2:** Systemic complaints based on sex, age and ethnicity of the outpatients at primary and tertiary care OPDs

Characteristic		Systemic complaint					
		Primary Care OPD (PC)			Tertiary Care OPD (TC)		
		The commonest symptom (%)	2nd commonest symptom (%)	3rd commonest symptom (%)	The commonest symptom (%)	2nd commonest symptom (%)	3rd commonest symptom (%)
Sex	Male	Respiratory (27%)	Dermatological (21.2%)	Musculo-skeletal (16.1%)	Dermatological (37.9%)	Respiratory (16.6%)	Musculo-skeletal (15.9%)
	Female	Respiratory (35.8%)	Musculo-skeletal (16.7%)	Dermatological (12.1%)	Dermatological (25.8%)	Respiratory (20.8%)	Musculo-skeletal (15.4%)
Age	Young	Respiratory (58.3%)	Eye related (12.5%)	Constitutional (8.3%)	Dermatological (39.5%)	Respiratory (21.1%)	Gastrointestinal (10.5%)
	Adult	Respiratory (44.6%)	Dermatological (12.2%)	Musculo skeletal (9.5%)	Dermatological (30.1%)	Respiratory (20.5%)	Musculo skeletal (11%)
	Middle-aged	Respiratory (33.8%)	Dermatological (12.8%)	Musculo skeletal (12.0%)	Dermatological (30.1%)	Respiratory (16.7%)	Musculo skeletal (16%)
	Older persons	Musculo skeletal (28.1%)	Dermatological (23.1%)	Respiratory. (18.2%)	Dermatological (28%)	Musculo skeletal (22.9%)	Respiratory (21.2%)
Ethnicity	Sinhalese	Respiratory (32.5%)	Dermatological (15.8%)	Musculo-skeletal (15.8%)	Dermatological (29.5%)	Respiratory (18%)	Musculo-skeletal (16.2%)
	Tamil	Respiratory (33.3%)	Musculo-skeletal (33.3%)	Dermatological (8.3%)	Dermatological (34.5%)	Respiratory (24.1%)	Musculo- skeletal (10.3%)
	Muslim	Respiratory (30.4%)	Musculo- skeletal (17.4%)	Dermatological (15.2%)	Dermatological (41.2%)	Respiratory (35.3%)	Musculo- skeletal (11.8%)

### Comparison of morbidities

The percentage of outpatients with respiratory symptoms was significantly higher at primary care OPD (32.4%) than at tertiary care (19.2%) (χ2 = 16.773, *p* = 0.00042). The percentage of outpatients with dermatological symptoms was significantly higher at the tertiary care OPD (30.4%) than at the primary care OPD (15.6%) (χ2 = 22.404, *p* = 0.00001). The proportions of musculo-skeletal, neurological, gastro-intestinal, constitutional and cardio-vascular symptoms were not significantly different between the two OPDs (*p* > 0.05).

### Comorbidities

The prevalence of self-reported comorbidities (NCDs) among outpatients at the tertiary care OPD (37.9%, 95% CI: 33.2–42.8) was higher compared to primary care (33.2%, 95% CI: 28.5–38.3). However, as denoted by overlapping confidence intervals, this difference was not statistically significant (χ2 = 1.75, *p* = 0.185).

**Fig. 2:**
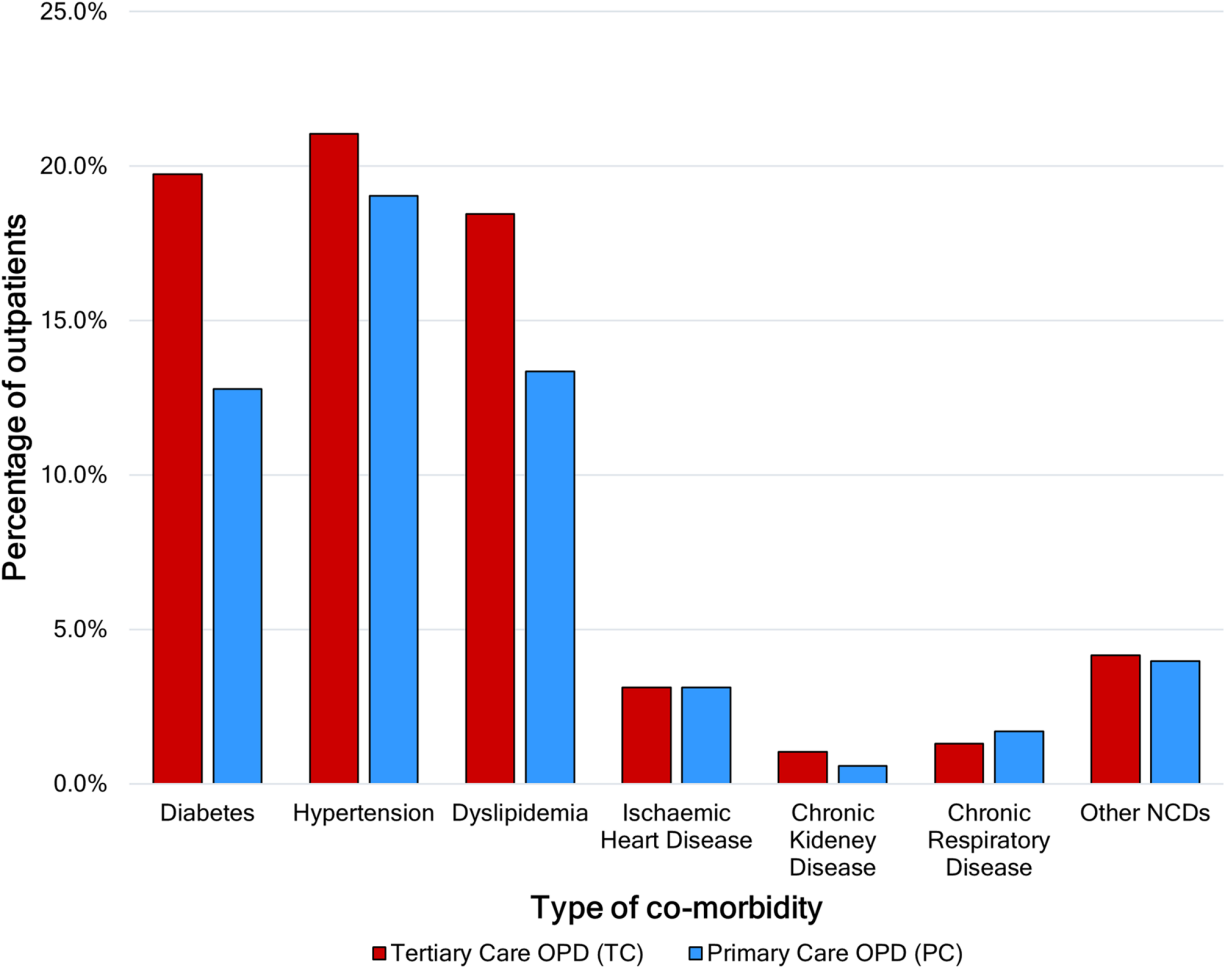
Self-reported comorbidities of outpatients at primary and tertiary care OPDs

As illustrated in Fig. 2, hypertension was the highest self-reported comorbidity by outpatients in both settings, whereas chronic kidney disease was the least reported. Overall, the prevalence of comorbidities at the tertiary care OPD was higher compared to primary care. However, these differences were not statistically significant except in diabetes. 19.7% of outpatients reported having diabetes in tertiary care compared to 12.8% in primary care (χ2 = 6.84, *p* = 0.01). Malignancies, rheumatological disorders and degenerative disorders were the main categories under other NCDs.

### Multimorbidity

The prevalence of multimorbidity at the tertiary care OPD (19.0%, 95% CI: 15.3–23.1) was higher than that at primary care (15.9%, 95% CI: 12.4–20.0). This difference in multimorbidity was not statistically significant (χ2 = 1.18, *p* = 0.276).

At both OPDs, females had a higher prevalence of multimorbidity than males (19.1% vs. 10.9% at PC and 19.2% vs. 18.6% at TC). Nevertheless, this difference was statistically significant only in primary care (χ2 = 4.125, *p* = 0.0423). At both OPDs, multimorbidity increased with the age of outpatients, with the highest multimorbidity observed among older persons (32.2% at PC and 39.8% at TC). Sinhalese outpatients had the highest multimorbidity at primary care OPD (18.5%), followed by the Tamils (8.3%) and the Muslims (2.2%). At the tertiary care OPD, multimorbidity was predominantly seen among Muslim outpatients (35.3%), followed by the Sinhalese (18.3%) and the Tamils (17.2%).

### Prescribing patterns

Out of the 737 participants who were recruited for the study at both settings, prescriptions could be traced at the OPD pharmacies only for 647 participants (88%).

### Medicines per encounter

The mean number of medicines per encounter at the primary care OPD was 3.86 (95% CI: 3.73–3.99), and the mean number of medicines per encounter at the tertiary care OPD was 3.47 (95% CI: 3.31–3.63). The median number of medicines per encounter at the primary care OPD was 4 (IQ range: 2), and the median number of medicines per encounter at the tertiary care OPD was 3 (IQ range: 1). The distribution of the number of medicines per encounter (discrete data) at each OPD showed a non-normal distribution (Kolmogorov‒Smirnov test: *p* < 0.05). The difference in the number of medicines per encounter between the OPDs was statistically significant (U = 30120.5, *p* < 0.001).

### Medicines per encounter by morbidity

As shown in Table 3, in the primary care setting, patients with constitutional symptoms, respiratory symptoms and musculo-skeletal symptoms were prescribed the highest number of medicines per encounter. Similarly, in the tertiary care setting, the highest number of medicines per encounter was also for constitutional symptoms and respiratory symptoms, followed by cardio-vascular symptoms. At both OPDs, when considering morbidities that exceeded over ten encounters, the least number of medicines per consultation was prescribed for dermatological symptoms. As seen in Table 3, despite encounters no medicines were prescribed for certain complaints like menstrual symptoms and pregnancy planning.

When considering the majority of systemic complaints, a higher number of medicines per encounter was prescribed at the primary care OPD facility. Nevertheless, this pattern was reversed for dermatological and genito-urinary symptoms.

**Table 3:** Medicines per encounter by the type of systemic complaint between primary and tertiary care OPDs

Type of systemic complaint	Primary Care OPD (PC)			Tertiary Care OPD (TC)		
	No. of encounters	Mean no. of medicines per encounter	Median no. of medicines per encounter	No. of encounters	Mean no. of medicines per encounter	Median no. of medicines per encounter
Respiratory symptoms	103	4.18	4	71	3.80	4
Dermatological symptoms	33	2.58	3	113	3.01	3
Musculo-skeletal symptoms	55	4.15	4	56	3.52	3
Neurological symptoms	35	3.63	4	43	3.26	3
Gastro-intestinal symptoms	14	3.64	4	22	3.55	3
Constitutional symptoms	20	4.45	5	15	3.86	4
Cardio-vascular symptoms	12	3.92	4	13	3.80	3
Genito-urinary symptoms	6	3.00	3	4	3.33	3
Eye-related symptoms	3	3.67	4	7	3.50	4
For investigations	1	2.00	2	4	4.00	4
Ear-related symptoms	1	5.00	5	5	2.00	2
Oral-cavity related symptoms	0	.	.	2	2.00	2
For referrals	1	2.00	2	3	.	.
For vaccination	0	.	.	1	3.00	3
Menstrual symptoms	0	.	.	2	.	.
Psychological symptoms	0	.	.	2	6.00	6
For planning pregnancy	0	.	.	1	.	.

### Types of medicine

As shown in Table 4, among the 284 outpatients at PC, the most prescribed type of medicine was paracetamol (57.4%), followed by chlorpheniramine (55.3%), dexamethasone (55.3%), vitamin C (33.5%) and amoxicillin (25%).

Among the 364 outpatients at TC, the first and second most prescribed types of medicine were also paracetamol (38.1%) and chlorpheniramine (31.9%). This was followed by omeprazole (31.3%), diclofenac sodium (23.4%) and dexamethasone (14.8%).

The three most common antibiotics encountered by outpatients at the primary care OPD were amoxicillin (25%), metronidazole (3.6%) and nitrofurantoin (1.7%), and at the tertiary care OPD, they were amoxicillin (13.7%), cephalexin (6.3%) and metronidazole (2.8%).

**Table 4:** The most prescribed types of medicines at primary and tertiary care OPDs

Type of medicine	Primary Care OPD (PC) (Total = 284)		Tertiary Care OPD (TC) (Total = 364)	
	Number of outpatients	Percentage (%)	Number of outpatients	Percentage (%)
Paracetamol	163	57.4	139	38.2
Chlorpheniramine	157	55.3	116	31.9
Dexamethasone	157	55.3	54	14.8
Omeprazole	60	21.1	114	31.3
Vitamin C	95	33.5	47	12.9
Diclofenac sodium	50	17.6	85	23.4
Amoxicillin	71	25.0	50	13.7
Famotidine	60	21.1	39	10.7
Salbutamol	48	16.9	48	13.2
Theophylline	69	24.3	1	0.3
Domperidone	19	6.7	46	12.6
Vitamin B Complex	23	8.0	30	8.2
Prednisolone	17	6.0	27	7.4

### Categories of medicine

**Fig. 3:**
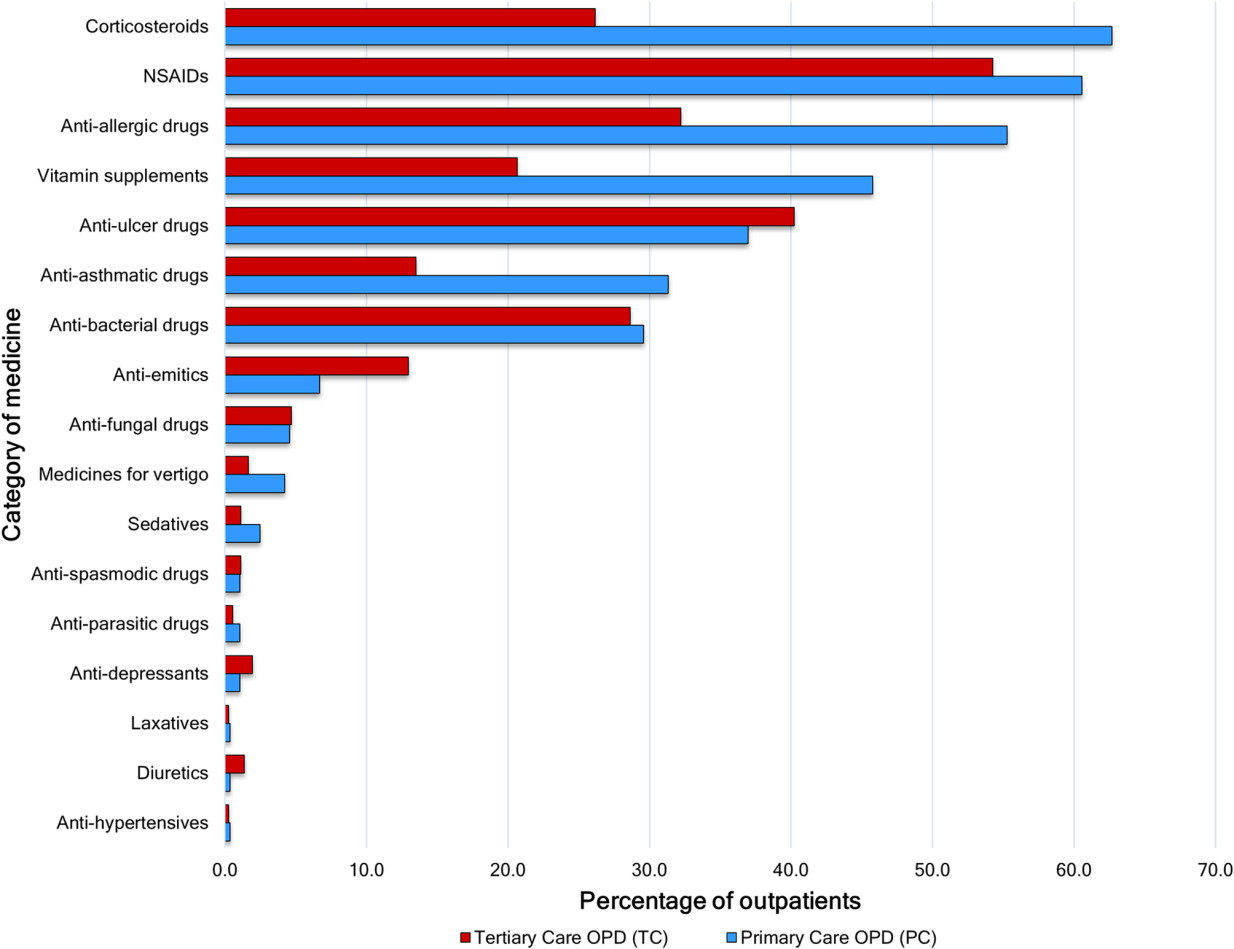
Categories of medicine prescribed at the primary and tertiary care OPDs

As illustrated in Fig. 3, the most prescribed category of medicine among outpatients at the primary care OPD was corticosteroids (62.7%), and NSAIDs were the second most prescribed category of medicine (60.6%). Anti-allergic drugs (55.3%), vitamin supplements (45.8%) and anti-ulcer drugs (37.0%) were the other commonly prescribed categories.

Among outpatients at the tertiary care OPD, the most prescribed category of medicine was NSAIDs (54.3%), followed by anti-ulcer drugs (40.2%), anti-allergic drugs (32.2%), anti-bacterial drugs (28.7%) and corticosteroids (26.2%).

Table 5 shows the differences in commonly prescribed categories of medicines between primary and tertiary care OPDs with their statistical significance. There were statistically significant differences in the prescribing of corticosteroids, anti-allergic, anti-asthmatic, anti-emetic drugs and vitamin supplements. However, there were no significant differences between the prescribing of NSAIDs, anti-fungal and anti-ulcer medications. The percentage of antibiotics encountered at the primary care OPD (29.6%) was higher than that at the tertiary care OPD (28.7%), but this difference was not statistically significant.

**Table 5:** Differences in prescribed medicine categories between the primary and tertiary care OPDs and their statistical significance

Category of medicine	Primary Care OPD (PC) (Total = 284)		Tertiary Care OPD (TC) (Total = 364)		Test statistic and significance
	Number of outpatients	Percentage (%)	Number of outpatients	Percentage (%)	
NSAIDs	172	60.6	197	54.3	χ2 = 2.57, *p* = 0.108
Corticosteroids	178	62.7	95	26.2	χ2 = 87.05, *p* < 0.00001
Anti-allergic drugs	157	55.3	117	32.2	χ2 = 35.18, *p <* 0.00001
Anti-asthmatic drugs	89	31.3	49	13.5	χ2 = 30.21, *p <* 0.00001
Vitamin supplements	130	45.8	75	20.7	χ2 = 46.42, *p <* 0.0001
Anti-bacterial drugs	84	29.6	104	28.7	χ2 = 0.06, *p* = 0.796
Anti-fungal drugs	13	4.6	17	4.7	χ2 = 0.003, *p* = 0.955
Anti-ulcer drugs	105	37.0	146	40.2	χ2 = 0.70, *p* = 0.400
Anti-emetic drugs	19	6.7	47	12.9	χ2 = 6.81, *p* = 0.009

### Categories of medicine by morbidity

Table 6 illustrates the breakdown of categories of medicine prescribed at the primary and tertiary care OPDs by the type of systemic complaint.

There were statistically significant differences in the prescription of corticosteroids among outpatients with respiratory, dermatological, musculo-skeletal and constitutional symptoms between the two settings. These differences followed a similar pattern to the total prescription of corticosteroids where the primary care OPD had a higher proportion than the tertiary care. Statistically significant differences where the primary care setting had greater proportions were also noted in the prescription of anti-allergic and anti-asthmatic drugs for outpatients with respiratory symptoms and in the prescription of vitamin supplements for outpatients with musculo-skeletal and dermatological symptoms.

Meanwhile, contrary to the overall difference, more antibiotics were administered for outpatients with respiratory symptoms at the tertiary care OPD (63.4%) compared to the primary care (35.0%), and this difference was statistically significant. The differences observed for each systemic complaint under categories of NSAIDs, anti-fungal, anti-ulcer and anti-emetic drugs were not statistically significant.

**Table 6:** Counts and percentages of outpatients prescribed with specific categories of medicine by the type of systemic complaint

Type of systemic complaint/ symptom	Counts and percentages of outpatients prescribed with specific categories of medicine																																			
	NSAIDs				Corticosteroids				Anti-allergic drugs				Anti-asthmatic drugs				Vitamin supplements				Anti-bacterial drugs				Anti-fungal drugs				Anti-ulcer drugs				Anti-emetic drugs			
	PC		TC		PC		TC		PC		TC		PC		TC		PC		TC		PC		TC		PC		TC		PC		TC		PC		TC	
	*n*	%	*n*	%	*n*	%	*n*	%	*n*	%	*n*	%	*n*	%	*n*	%	*n*	%	*n*	%	*n*	%	*n*	%	*n*	%	*n*	%	*n*	%	*n*	%	*n*	%	*n*	%
Respiratory	45	43.7	40	56.3	80	77.7^b^	32	45.1^b^	98	95.1^b^	53	74.6^b^	68	66.0^a^	34	47.9 ^a^	53	51.5	30	42.3	36	35.0^b^	45	63.4^b^	1	1	1	1.4	13	12.6	17	23.9	4	3.9	6	8.5
Dermatological	12	36.4	29	25.7	13	39.4 ^a^	25	22.1 ^a^	12	36.4	29	25.7	2	6.1	3	2.7	14	42.4 ^b^	7	6.2 ^b^	10	30.3	31	27.4	7	21.2	14	12.4	6	18.2	19	16.8	0	0	3	2.7
Musculo-skeletal	50	90.9	55	98.2	39	70.9^b^	20	35.7^b^	9	16.4	5	8.9	4	7.3	1	1.8	28	50.9 ^a^	15	26.8 ^a^	5	9.1	7	12.5	2	3.6	2	3.6	42	76.4	48	85.7	2	3.6	5	8.9
Neurological	27	77.1	33	76.7	16	45.7	8	18.6	11	31.4	9	20.9	3	8.6	2	4.7	13	37.1	8	18.6	6	17.1	2	4.7	2	5.7	0	0	19	54.3	21	48.8	2	5.7	7	16.3
Gastro-intestinal	6	42.9	10	45.5	4	28.6	2	9.1	4	28.6	2	9.1	1	7.1	1	4.5	2	14.3	5	22.7	4	28.6	5	22.7	1	7.1	0	0	11	78.6	19	86.4	6	42.9	15	68.2
Constitutional	14	70	11	73.3	15	75.0 ^a^	3	20.0 ^a^	19	95	12	80	10	50	5	33.3	10	50	5	33.3	14	70	7	46.7	0	0	0	0	2	10	3	20	1	5	2	13.3
Cardio-vascular	10	83.3	10	76.9	7	58.3	2	15.4	1	8.3	1	7.7	1	8.3	1	7.7	3	25	1	7.7	2	16.7	2	15.4	0	0	0	0	10	83.3	10	76.9	3	25	4	30.8
Genito-urinary	3	50	2	50	0	0	0	0	1	16.7	0	0	0	0	0	0	5	83.3	2	50	5	83.3	2	50	0	0	0	0	2	33.3	2	50	0	0	2	50
Eye-related	2	66.7	1	14.3	3	100	1	14.3	2	66.7	2	28.6	0	0	0	0	2	66.7	0	0	1	33.3	0	0	0	0	0	0	0	0	1	14.3	0	0	1	14.3
Ear-related	1	100	1	20	1	100	0	0	0	0	0	0	0	0	0	0	0	0	0	0	1	100	1	20	0	0	0	0	0	0	0	0	1	100	0	0

n – count, % - percentage, ^a^ – statistically significant difference in proportions that is less than 0.05, ^b^ – statistically significant difference in proportions that is less than 0.001

## Discussion

To the best of our knowledge, this was the first comparative study in a South Asian setting that assessed both the morbidity profile and pharmaceutical management of adult outpatients between primary and tertiary care levels. The majority of the sample were females and were middle-aged. The two cohorts did not differ in sex or mean age. The duration of presenting symptoms was higher in tertiary care OPD than in primary care. The most common systemic complaint in primary care OPD was respiratory symptoms, whereas it was dermatological symptoms in tertiary care. Other systemic complaints were not significantly different between the two OPDs. The pattern of the most common systemic complaint did not differ by sex, age group or ethnicity at either OPD, with the exception of musculo-skeletal symptoms being the most common complaint among older persons in primary care. Psychological symptoms were less than 1% among outpatients at both OPDs. The prevalence of comorbidities at the tertiary care OPD was higher compared to primary care, but these differences were not statistically significant except in diabetes. There was no statistically significant difference in multimorbidity between the two OPDs. The number of medicines per encounter was higher in primary care OPD than in tertiary care. Most medicines per encounter were prescribed for constitutional and respiratory symptoms in both OPDs. The most prescribed type of medicine in both settings was paracetamol. The most prescribed category of medicine in the primary care OPD was corticosteroids, while in tertiary care it was NSAIDs. Prescribing corticosteroids, anti-allergic drugs, vitamin supplements and anti-asthmatic drugs was significantly higher in the primary care OPD, whereas prescribing anti-emetic drugs was significantly higher in tertiary care. When the categories of medicines were disaggregated by the type of systemic complaint, the difference between the settings largely followed these overall differences. The proportion of antibiotics encountered at the primary care OPD was higher than in tertiary care, but this difference was not statistically significant.

### Sociodemographic profile

As seen in Table 1, the outpatient populations in the two OPDs did not differ in age or sex composition. Similar observations have been made in comparisons between primary and tertiary care in Sri Lanka [25]. Female predominance was observed in both outpatient settings. This is a commonly observed phenomenon in Sri Lanka, in the region and the world [8, 9, 26]. This could be explained by better help-seeking behaviours among females and also by the ‘male health survival paradox’, where males report better health than females but face higher mortality [27, 28]. At both the primary and tertiary levels, nearly 1 out of 3 outpatients were over 60 years of age, indicating the impact of the demographic transition on the health system of Sri Lanka [11]. Differences in the ethnic composition of minorities at the two OPDs tallied the racial distribution in the drainage areas, where PC had more Muslim residents and where TC attracted more Tamil-speaking outpatients.

### Morbidity profile

Respiratory symptoms being the most common complaint in primary care OPDs has been previously observed in private sector OPDs in Sri Lanka [29]. These findings are also consistent with Sri Lankan morbidity patterns in Global Burden of Diseases (GBD) studies 2019, where lower respiratory tract infections and chronic obstructive pulmonary disease were among the top ten causes of morbidity [30]. Our findings differed from previous outpatient studies in tertiary care in Sri Lanka, where musculo-skeletal symptoms were the most common [9, 21]. However, dermatological symptoms have been common in tertiary care OPDs in other regional studies [31, 32].

In South Asia, outpatient morbidities vary by age, sex and ethnicity [9, 33]. Further research is needed to identify the reasons for this lack of moderation of morbidity by sociodemographic variables in our study. However, musculo-skeletal symptoms were seen to increase with the age of outpatients at the primary care OPD, and this has been reported before [26]. Psychological symptoms being less than 1% in both settings may reflect poor help-seeking behaviours for mental health problems and somatisation of psychological morbidities rather than an actual low prevalence [34].

The difference in the duration of symptoms could be partially explained by the most common systemic complaint at each OPD, where respiratory symptoms are usually acute in their course and dermatological conditions are largely chronic presentations. However, findings related to chronic presentations in tertiary care need to be viewed with caution. Given the improved health access, pluralistic health system and bypassing behaviours of outpatients in Sri Lanka, these encounters could have been revisits to the health provider, an aspect that was not explored by this study.

The overall differences in the morbidity profile between primary and tertiary care OPDs cannot be attributed to age or sex, as those variables did not significantly differ between the settings. Auxiliary services that are offered at the tertiary care OPD, such as the availability of superior surgical care including an OPD theatre and the ability to be admitted to a dermatology ward, could have attracted more outpatients with dermatological symptoms. Another reason could have been the wider range of medicines only available at the tertiary care OPD pharmacies, including mixtures of topical applications for dermatological complaints, whereas the majority of commonly used medicines for respiratory symptoms are readily available in primary care OPDs. These morbidity characteristics could also define the cohort of outpatients who tend to bypass primary care.

The prevalence of self-reported diabetes, hypertension and dyslipidemia at the tertiary care OPD largely tallied with the NCD burden in Sri Lanka as reported by the WHO STEPS Survey and other national surveys [35, 36]. Diabetes and hypertension are also among the most common chronic conditions among outpatients in South Asia [37]. However, further research is needed to identify whether the relatively higher prevalence of diabetes in tertiary care is a feature of the cohort of outpatients who have bypassed primary care hoping for better NCD-integrated health services. In Sri Lanka, outpatients who receive NCD treatment from specialised clinics at tertiary care are known to visit the OPD of the same hospital to get treated for other minor ailments during the same visit, which could in part explain the above findings. The low self-reported prevalence of ischaemic heart disease, chronic obstructive pulmonary disease and chronic kidney disease compared to the national level burden also needs further investigation [35]. These differences may have ensued from a self-reporting information bias rather than an actual district-level variation of these morbidities.

Higher multimorbidity in tertiary care may have arisen due to the same reasons that led to higher comorbidities and need further assessment. However, compared to the global estimates of multimorbidity in primary care, this study shows a markedly lower prevalence of 15% [38]. Associations of multimorbidity detected by this study, such as female sex and increasing age, are supported by regional evidence in South Asia [37]. More evidence should be generated in Sri Lanka on the ethnic variations in multimorbidity observed in this study.

### Pharmaceutical management

The average number of medicines per encounter is a core prescribing indicator in outpatient care. This was higher than the World Health Organization’s recommended value (1.6–1.8) in both primary and tertiary care OPDs but lower than that level considered as the cut-off level for polypharmacy (4) [39]. The average number of medicines per encounter at primary care OPD was higher than the values observed before in Sri Lanka and in the region [10, 17]. This observation is counterintuitive as you would expect more medicines to be prescribed at the level where there was a higher multimorbidity. Medicines per encounter being the highest for constitutional symptoms in both settings indicates the need for clinical guidance on managing such outpatient presentations. Encounters shown in Table 3 where no medicines were prescribed were likely referred for further care.

Previous studies conducted at both primary and tertiary care OPDs in Sri Lanka and in the region have shown paracetamol to be the most prescribed type of drug [31, 40, 41]. NSAIDs being prescribed for over half of outpatients at each setting tallied the morbidity patterns where, as seen in Table 6, they were mainly prescribed for musculoskeletal and respiratory symptoms. Increased prescription of anti-ulcer drugs at tertiary care OPDs has been noted in regional studies before and as shown in Table 6 it accompanied the prescription of NSAIDs [31].

Every one in two patients being prescribed corticosteroids at the primary care OPD was not compatible with its morbidity profile. As illustrated in Table 6, corticosteroids were prescribed in higher counts for musculo-skeletal, neurological and constitutional symptoms in addition to the targeted respiratory symptoms. Misuse of oral corticosteroids in primary care has been previously reported in South Asia [19]. In Sri Lanka, oral corticosteroids are alleged to be misused in primary care settings for the rapid recovery of respiratory tract infections, but the literature is scarce in this regard. This study provides strong evidence on the need for future research assessing the rational prescription of oral corticosteroids in primary care.

Higher prescribing of anti-allergic and anti-asthmatic drugs at the primary care OPD, as shown in Table 6, was mainly for outpatients with respiratory symptoms and was compatible with their morbidity profile. However, this was not the case for multivitamin supplements. Multivitamins, especially Vitamin B complex, are frequently misused in South Asian outpatient care [18]. In Sri Lankan outpatient settings, vitamin supplements are often prescribed as a part of a cocktail rather than on a scientific basis to appease patients who seek pills from allopathic healthcare providers. There is currently no national guideline for the prescription of these supplements in outpatient care. Nearly one in two patients were prescribed vitamin supplements in the primary care OPD and as evident in Table 6 they were mainly for respiratory and musculo-skeletal symptoms. Both technical and policy interventions are required to address this issue of irrational- and over-usage. Nevertheless, contrary to the current observations, more multivitamins were prescribed in tertiary care than in primary care in a regional study [42].

Although the percentage of antibiotics encountered in primary care was slightly higher, both OPDs were below the WHO-recommended cut-off for outpatient settings (< 30%) [43]. The percentage of antibiotics issued at the primary care OPD was significantly lower compared to pooled estimates for LMICs (55%), even with respiratory symptoms predominating its morbidity profile. The percentage of antibiotics dispensed at the tertiary care OPD was also smaller than the proportions seen among regional counterparts [40, 44]. Previous studies on antibiotic prescription in tertiary care in Sri Lanka have given mixed results [40, 45]. Therefore, more evidence is needed to conclude whether Sri Lanka may be performing better in antibiotic prescription at the primary and tertiary care levels than other South Asian countries. Amoxicillin has been the most prescribed antibiotic among other outpatient studies conducted in state-sector tertiary care in Sri Lanka [45, 46].

### Limitations

Although this study had strengths in generating primary evidence from a randomly selected, large sample to compare the attributes of primary and tertiary care OPDs in an LMIC health system that had no outpatient surveillance at present, it also had several major limitations. First, though the comparison of two facilities within the same district and time point strengthened the internal validity of the study, it may have significantly limited its generalisability. The ethnic composition of the study population also limited the generalisability of the study to specific regions in Sri Lanka. Second, the reduction in the sample size in subsequent analyses due to missing prescriptions may have affected the power of the study. These prescriptions were likely to be missing due to outpatients visiting private sector pharmacies, being referred to other specialised units or due to alternative reasons for encounters like visiting to get investigations done. Other nonsignificant results in the comparison of smaller subcategories may also have been due to limited power. Third, since the questionnaire was deliberately made concise to be easily administered in a busy OPD setting, it did not collect information on important confounders such as treatment history, income, occupation and education. Failure to apply multivariate analyses by including such covariates was another major limitation. Fourth, self-reporting of morbidities may have added a significant recall bias to the findings of the study. Some patients were assisted in filling out the questionnaire due to problems with vision, which may have affected the uniformity of data collection. Fifth, we were unable to use consistent selection criteria like similar admission rates for the two settings that were being compared. This was due to inherent disparities in utilization between these settings that have been created due to the bypassing phenomena.

## Conclusions

On balance, the presenting morbidities of the primary care OPD differed from those of the tertiary care in terms of duration and type. However, most comorbidities and the multimorbidity of outpatients did not differ between the two settings. Pharmaceutical management also varied between the two OPDs in terms of medicines per encounter and prescribed categories.

As recommendations for practice, we encourage local health administrators to plan OPD services and resources to match the morbidities and comorbidities identified by this study. The prevalence of older age, NCDs and multimorbidity that was largely similar between primary and tertiary levels suggests the need to incorporate geriatric care and NCD-related capacity building into outpatient services across the health system. We also recommend increasing the focus on limited disclosure of psychological morbidities by outpatients when training OPD staff. Clinical guidance on prescribing for patients presenting with constitutional symptoms can be recommended. We also recommend that national-level health planners investigate the prescription of oral corticosteroids and multivitamin supplements in primary care outpatient settings in Sri Lanka and formulate necessary guidelines. These should include educational interventions for healthcare staff and clinical guidance on prescribing oral corticosteroids for common respiratory symptoms in outpatient settings. Given the gaps in knowledge, similar directions can also be suggested for regional and global research communities to look into rational prescribing of these commonly prescribed drugs in OPDs. Further studies are needed to test the hypotheses generated by this study on the patterns of NCDs, multimorbidity, polypharmacy and other management practices between primary and tertiary care outpatient settings and to assess their relationship with bypassing behaviours.

### Electronic supplementary material

Below is the link to the electronic supplementary material.

Supplementary Material 1

Supplementary Material 2

## Data Availability

No datasets were generated or analysed during the current study.
