# Supplementary material for: Morbidity profile and pharmaceutical management of adult outpatients between primary and tertiary care levels in Sri Lanka: a dual-centre, comparative study
Source: BMC Prim Care. 2024 Jun 6;25:200. doi: 10.1186/s12875-024-02448-8 (PMC11155019; doi:10.1186/s12875-024-02448-8)
Supplement: Supplementary file 1 — Supplementary Material 1 [file 12875_2024_2448_MOESM1_ESM.pdf]

"Morbidity profile and pharmaceutical management of outpatients in primary and tertiary levels in Sri Lanka": Questionnaire

Please write the answer in the box provided.

1) OPD Registration No.: ☐

2) Age (in years) : ☐

3) Resident area : ☐

Please tick the relevant box.

4) Sex : Male ☐ Other ☐
   Female ☐ Do not wish to declare ☐

5) Ethnicity: Sinhala ☐ Other ☐
   Tamil ☐ Do not wish to declare ☐
   Muslim ☐
   Burgher ☐

6) Please select the **main reason** for visiting the OPD today. Please select **only one** presenting complaint. Please write the complaint under '**Other**' if unlisted.

1. For investigations ☐
2. For vaccination ☐
3. Body aches ☐
4. Fever ☐
5. Tiredness ☐
6. Abdominal pain ☐
7. Abdominal distension ☐
8. Vomiting ☐
9. Diarrhoea ☐
10. Constipation ☐
11. Change in bowel habits ☐
12. Toothache ☐
13. Complaint related to eyes ☐
14. Complaint related to ears ☐
15. Chest pain ☐
16. Palpitations ☐
17. Back pain ☐
18. Joint pain ☐
19. Headache ☐
20. Dizziness ☐
21. Sensory loss in limbs ☐
22. Weakness in limbs ☐
23. Feeling fear ☐
24. Feeling depressed ☐
25. Difficulty in breathing ☐
26. Wheeze ☐
27. Cough ☐
28. Cold ☐
29. Sore throat ☐
30. Complaint related to nose ☐
31. Complaint related to skin ☐
32. Wound ☐
33. Animal bite ☐
34. Blunt injury ☐
35. Cut injury ☐
36. Complaint related to nails ☐
37. Dysuria ☐
38. Change in urinary habits ☐
39. Complaint in menstruation ☐
40. Other ☐

7) Duration of the presenting symptom: ☐ (days/weeks/months)

Please tick the relevant box if you have been diagnosed with **any** of the following non-communicable diseases. You can tick more than one box. Please write the condition under **‘Other’** if unlisted.

8) Diabetes ☐ Chronic kidney disease ☐

Hypertension ☐ Chronic lung disease ☐

Dyslipidemia ☐ Other ☐

Ischaemic heart disease ☐
