# Supplementary material for: Morbidity profile and pharmaceutical management of adult outpatients between primary and tertiary care levels in Sri Lanka: a dual-centre, comparative study
Source: BMC Prim Care. 2024 Jun 6;25:200. doi: 10.1186/s12875-024-02448-8 (PMC11155019; doi:10.1186/s12875-024-02448-8)
Supplement: Supplementary file 2 — Supplementary Material 2 [file 12875_2024_2448_MOESM2_ESM.pdf]

“Morbidity profile and pharmaceutical management of outpatients in primary and tertiary levels in Sri Lanka”: Datasheet

No.	OPD Registration No.	Name of the drug	Dosage	Frequency	Duration	Other remarks
01.						
02.						
03.						
04.						
